# Urinary Nephrin: A Potential Biomarker of Early Glomerular Injury in a Cohort of Pregnant Women Attending Routine Antenatal Care Services

**DOI:** 10.1155/2024/9089557

**Published:** 2024-11-02

**Authors:** Belete Biadgo Mesfine, Danica Vojisavljevic, Ranjna Kapoor, David Watson, Yogavijayan Kandasamy, Donna Rudd

**Affiliations:** ^1^College of Public Health, Medical and Veterinary Science, James Cook University, 1 James Cook Drive, Douglas, Townsville, Queensland 4811, Australia; ^2^College of Medicine and Health Sciences, School of Biomedical and Laboratory Sciences, Department of Clinical Chemistry, University of Gondar, Gondar, Ethiopia; ^3^Maternal Fetal Medicine Unit and Department of Obstetrics and Gynaecology, Townsville University Hospital, Townsville, Australia; ^4^Department of Neonatology, Townsville University Hospital, 100 Angus Smith Dr, Douglas, Queensland 4814, Townsville, Australia

## Abstract

**Introduction:** Glomerular injury may occur during pregnancy as a consequence of systemic disease and pregnancy-related medical complications. While urinary nephrin has been shown to provide early identification of preeclampsia (PE) in high-risk pregnancies, the role of urinary nephrin in determining glomerular injury in pregnant women is yet to be explored. This study aimed to investigate the use of urinary nephrin as a predictor for early glomerular injury in a study conducted at the Townville University Hospital.

**Methods and Materials:** A cross-sectional study was conducted. All pregnant women with a full dataset (*n* = 273) were classified into three categories according to their urinary albumin-to-creatinine ratio (ACR): normoalbuminuria, microalbuminuria and macroalbuminuria. Continuous variables were compared between groups, and the cut-off value for the urinary nephrin-to-creatinine ratio (NCR) was determined to predict albuminuria as an indirect indicator of early glomerular injury. The percentages of pregnant women who had elevated nephrinuria were calculated for each of the ACR categories.

**Results:** Urinary NCR positively correlated with urinary ACR (*r* = 0.29, *p* < 0.0001). Urinary NCR increased comparably in women with normoalbuminuria, microalbuminuria and macroalbuminuria. Using a cut-off value of 14 ng/mg, nephrinuria was detected in 65% of women with normoalbuminuria, 95% with microalbuminuria and 100% with macroalbuminuria. Of the normoalbuminuric women who had an elevated urinary NCR (> 14 ng/mg), 78% were diagnosed with a hypertensive disorder and 63% were diagnosed with diabetes in pregnancy. In women with PE, urinary NCR and ACR were significantly higher when compared to women who did not develop PE. The AUC of the ROC for urinary NCR was 0.74 (95% CI: 0.650–0.824), with a sensitivity of 97% and a specificity of 36% to predict glomerular injury and a sensitivity of 93% and specificity of 42% to predict glomerular injury of PE.

**Conclusion:** The study found that urinary NCR were elevated not only in women with micro- and macroalbuminuria but also in pregnant women with normoalbuminuria. Increased urinary NCR without increased urinary albumin may be associated with early glomerular injury. Urinary NCR may be a more sensitive marker than microalbuminuria to detect early glomerular injury in women with systemic disease and adverse pregnancy outcomes.

## 1. Introduction

Kidney disease is an important and increasing public health burden, often under recognised particularly in remote communities and low socioeconomic populations [1, 2]. Kidney injury prior to or during pregnancy can accelerate the decline in maternal renal function and lead to adverse pregnancy outcomes [3]. While globally the incidence of acute kidney injury (AKI) in pregnancy has declined, concern remains for women in both developing and developed nations [4, 5].

Pregnancy-induced changes in glomerular filtration such as renal vasodilation, hyperfiltration and enhanced glomerular permeability occur in normal pregnancy, which can lead to a reduction in maternal serum creatinine (SCr) [6], making the early diagnosis of renal damage during pregnancy difficult and preexisting renal disease often underdiagnosed. Up to 5% of Australian women of childbearing age have increased markers of kidney disease [2], secondary to systemic disorders, including diabetes mellitus (DM) and/or primary hypertension (HTN). Pre-existing undiagnosed kidney disease coupled with damage secondary to pregnancy-related disorders (hypertensive or microangiopathic disorders) can lead to pregnancy-associated AKI (pr-AKI) [7]. In particular, preeclampsia (PE), a disease of the glomerulus during pregnancy, may further damage the glomerulus [8] and increase the risk of AKI [5]. Pr-AKI has been associated with a chronic decline in renal function with a recent case report of nephropathy during pregnancy progressing to end-stage kidney disease (ESKD) within 1 year postpartum [9].

Due to the bidirectional relationship between the placenta and the kidney, pregnancy is an opportune time to screen for renal disease [10]. The consequences of glomerular damage prior to and during pregnancy, and accompanying complications, mean that early detection and management of glomerular injury is vital. To date, routine chemical urinalysis and measurement of proteinuria are the main tools for diagnosis and monitoring of kidney disease progression [11]. The Kidney Disease Improving Global Outcome (KDIGO) guidelines for kidney disease assessment during pregnancy often incorporated into local hospital guidelines recommend urinary protein loss be monitored by measuring urinary albumin-to-creatinine ratio (ACR), as an indicator of progressive glomerular injury [11]. Measurement of urine albumin concentration using 24-h urine samples is recommended, but it is not feasible in actual clinical practice. Therefore, a spot urine sample for ACR analysis is commonly used [11]. KDIGO guidelines recommend an ACR level > 3 mg/mmol to classify patients as at a higher risk of glomerular injury, and this is used for monitoring the progression and staging of chronic kidney disease (CKD) [11]. New-onset HTN and proteinuria predicted by an ACR > 8 mg/mmol after 20 weeks of gestation correlated with maternal kidney damage from PE [3].

While both proteinuria and albuminuria are important for detecting patients with severe glomerular damage, a specific and sensitive diagnostic marker for the diagnosis of early glomerular injury may be of value. There has been increased interest in evaluating biomarkers for the early detection of glomerular damage [12–14]. Podocyte proteins have previously been demonstrated as useful biomarkers for predicting glomerular injury [15, 16]. In the past decade, it has been shown that urinary nephrin may provide a sensitive and specific marker of early glomerular injury preceding albuminuria [13, 17]. Nephrin is a structural and functional component of glomerular podocytes [18], and together with glomerular endothelial cells, the glomerular basement membrane (GBM) forms the glomerular filtration barrier, which allows the passing of the ultrafiltrate by restricting macromolecules. Preclinical and clinical studies have shown that nephrin expression decreased in proteinuric disease [19] and samples obtained from pregnant women with PE [20, 21]. An increased concentration of nephrin in urine is reported in glomerular injury [17], indicating damage to the glomerular podocytes. Early diagnosis and identification of new-onset maternal glomerular injury and management of pregnancy-related complications in a patient-centred approach could provide potential intervention strategies for the kidney health of the mother and foetus. As urinary ACR does not become elevated until there is substantial glomerular damage, monitoring with ACR may delay early diagnosis of the glomerular damage.

To our knowledge, there is limited evidence for the use of urinary nephrin as a potential diagnostic marker for early glomerular injury in a cohort of pregnant women. Therefore, the overarching aim of this study was to explore the association of urinary nephrin concentrations with the development of pregnancy-associated complications known to involve the kidney. To address this, the aims of this study are to

• Explore the association of urinary nephrin and nephrin-to-creatinine ratio (NCR) with ACR and other parameters of maternal renal function.
• Explore the association of urinary nephrin and NCR with the diagnosis of diabetes (gestational diabetes [GDM], Type 1 diabetes [T1DM] and Type 2 diabetes [T2DM]) during pregnancy.
• Explore the association of urinary nephrin and NCR with the diagnosis of PE.
• Investigate the diagnostic accuracy of urinary nephrin and NCR for the prediction of early glomerular injury using albuminuria as an indirect indicator of glomerular injury.

## 2. Materials and Methods

### 2.1. Study Design and Setting

This cross-sectional study was embedded within the larger ‘The Relationship between Maternal Health and Infant Kidney Development and Function Study' funded by the National Health and Medical Research Council (NHMRC) (Application ID: APP1159616), which is a prospective, longitudinal cohort of mother–infant dyads from pregnancy up until the child is 2 years of age carried out at the Townsville University Hospital (TUH), Queensland.

### 2.2. Study Population

The Townsville Hospital and Health Services have more than 2400 births per year, and the region has 10,000 births annually. Approximately 20%–25% of babies born in this hospital are from the Indigenous Community. The study was open to all pregnant women receiving antenatal care at TUH, and during the recruitment period (August 2019 to August 2021), obstetric history and other pre-existing medical conditions, such as diabetes, HTN and kidney disease, were collected.

There were no exclusion criteria. Maternal blood and urine were collected for renal function testing serum creatinine (SCr), serum cystatin C (sCysC), urinary albumin, ACR urinary nephrin and NCR at the antenatal visit. The samples were then processed and stored until analysis at −80°C. The demographic data were collected from the participant's antenatal records including maternal health during the pregnancy including the history of HTN, PE, pre-pregnancy DM or GDM. All data and biomarker results were entered into a Research Electronic Data Capture (REDCap) database and exported and deidentified for analysis.

### 2.3. Association of Serum and Urine Markers of Renal Function Between Women With Normo-, Micro- and Macroalbuminuria

Once the data were collated, the women (*N* = 273) were classified into three groups based on the ACR mg/mmol values according to the KDIGO guideline [22]. Normoalbuminuria (Normo: ACR < 3 mg/mmol, *N* = 225), microalbuminuria (Micro: ACR 3–30 mg/mmol, *N* = 38) and macroalbuminuria (Macro: ACR > 30 mg/mmol, *N* = 10). The SCr, sCysC, urinary nephrin and NCR were compared between the three groups.

The normoalbuminuric group was further subdivided for analysis based on the reported comorbidities: NORM: women with ACR < 3 mg/mmol and no reported comorbidities and normal albumin with comorbidities and NCOM: women with ACR < 3 mg/mmol but with reported comorbidities.

The micro- and macroalbuminuric groups were combined and further subdivided for analysis based on the reported comorbidities: ALB: women with ACR ≥ 3 mg/mmol without comorbidities and ACOM: women with ACR > 3 mg/mmol with reported comorbidities.

### 2.4. Association of Urinary NCR With a Diagnosis of Diabetes or PE During Pregnancy

Women with reported comorbidities were classified into two groups according to their clinical notes: Group 1: hypertensive group (PE/HTN) including women reported to have PE and/or HTN and Group 2: diabetes in pregnancy (DIP), women reported to have GDM, T1DM and T2DM. Due to small numbers in the HTN and T1DM and T2DM groups, these groups were combined with others for analysis, and these groups were compared to a NORM group: women with ACR < 3 mg/mmol and no reported comorbidities and NCOM group: women with ACR < 3 mg/mmol but with reported comorbidities.

### 2.5. Comparison of Urinary Nephrin and ACR for the Prediction of PE and DIP

The diagnostic accuracy of urinary NCR for predicting elevated ACR levels was determined by comparing the urinary NCR for women in the complete cohort and between those with normal albuminuria (Normo) and a combined group of micro-and macroalbuminuria. To determine the predictive potential of urinary NCR and ACR for glomerular injury of PE, the women were also classified into the PE group (*N* = 26) and compared to the NORM group: women with ACR < 3 mg/mmol and no reported comorbidities (*N* = 135). The PE group was clinically confirmed cases of PE with denovo HTN, blood pressure > 140/90 mmHg and with/without proteinuria > 300 mg/day and other pathological changes after 20 weeks of gestation according to the American College of Obstetrics and Gynaecology (AGOC) definition [23]. The women were > 20 weeks of gestation at the time of sample collection, and the women included were in the second and third trimesters. Women with clinically confirmed PE were used to estimate the predictive potential of urinary nephrin, NCR and ACR for glomerular injury of PE.

### 2.6. Sample Collection and Biomarker Measurement

A venous blood sample was collected using a plain tube for the measurement of SCr and sCysC. SCr (μmol/L) and sCysC (mg/L) concentrations were measured spectrophotometrically using the automated Beckman Coulter biochemistry analyser (AU480, Australia). The clinical and demographic characteristics of the study participants were collected as part of the routine follow-up.

Urine samples were collected into a clean, leak-proof container without any preservatives. Urine samples were stored at −80°C until analysis. Once thawed at room temperature, urine samples were centrifuged at 300 (RPM) for 10 min. The urine albumin concentration was measured using a urinary albumin immunoturbidimetric assay (Beckman Australia), while the urine creatinine concentration was measured using a kinetic modification of the Jaffe reaction (Beckman Australia) using the automated Beckman Coulter Biochemistry analyser (AU480, Beckman Australia). The urinary nephrin concentration was measured using a human nephrin sandwich ELISA (Human NPHS1/Nephrin, LS-Bio [LS-F21185], Inc., USA). The urine samples were analysed in duplicate, the limit of detection of the kit was 0.16 ng/mL, and the samples with higher concentrations (> 10 ng/mL) were diluted from 1:10 to 1:100 using a sample/standard diluent supplied by the manufacturer. The concentration of urinary nephrin was calculated from the standard curve and reported as ng/mL. The precision of the duplicate measurements was calculated in our experimental data, and the interassay and intra-assay %CV values were ≤ 10. Urinary nephrin and albumin were adjusted by dividing to the urine creatinine concentration and described as NCR ng/mg and ACR mg/mmol.

### 2.7. Statistical Analysis

Statistical analysis was performed using IBM SPSS Version 28 and GraphPad Prism 9. The normality of the data was visually checked using a QQ plot and histogram and statistically using the Kolmogorov–Smirnov test and the skewness and kurtosis *Z* scores of the data distribution divided by their standard errors. Continuous data were presented as mean ± SD for normally distributed data or as the median and interquartile range (IQR: 25^th^–75^th^ percentile) for non-normally distributed data; categorical data were presented as number (percent) and mean ± SEM as appropriate. A Kruskal–Wallis H test with post hoc Bonferroni correction and Mann–Whitney U nonparametric tests were performed to determine the difference in continuous variables between groups. The maternal clinical characteristics and biochemical parameters were compared. The predictive potential of urinary NCR for early glomerular injury was tested and described by a binary logistic regression model with receiver operating characteristics (ROC) analysis. The maximum value of the Youden *J* index (sensitivity + specificity-1) and the area under the curve (AUC) with its 95% CI were used to estimate the cut-off value for urinary NCR (ng/mg). The diagnostic estimates (sensitivity, specificity, likelihood ratios and predictive values) were calculated for the optimal cut-off values of the biomarker in predicting early glomerular injury. Furthermore, the diagnostic performances of urinary nephrin, NCR and ACR in predicting adverse pregnancy outcomes (PE) were described by ROC analysis and AUC. Correlation was performed to determine the association of urinary NCR with other biochemical parameters. A *p* value of < 0.05 is considered statistically significant in all cases.

## 3. Results

### 3.1. Maternal Characteristics

**Figure 1:**
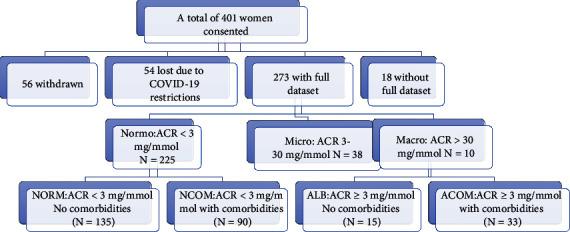
Flow diagram summarising the study participants: 273 women participated in the trial; subgroup analysis based on ACR levels; normoalbuminuria (Normo: ACR < 3 mg/mmol, (*N* = 225)), microalbuminuria (Micro: ACR 3–30 mg/mmol, (*N* = 38)) and macroalbuminuria (Macro: ACR > 30 mg/mmol, (*N* = 10)). The normoalbuminuric group was further divided into normal albumin group (NORM): women with ACR < 3 mg/mmol and no reported comorbidities, and a normal albumin with comorbidities (NCOM): women with ACR < 3 mg/mmol but with reported comorbidities. The micro- and macroalbuminuric groups were combined and further subdivided for analysis based on the reported comorbidities: ALB: women with ACR ≥ 3 mg/mmol without comorbidities and ACOM: women with ACR > 3 mg/mmol with reported comorbidities.

Of the 401 women who consented to this study, 56 women withdrew from the study, 54 did not attend antenatal clinics due to coronavirus disease 19 (COVID-19) restrictions, and 18 did not have a full dataset (Figure 1).

The maternal characteristics of the study population are summarised in Table 1. Overall, 273 pregnant women took part in this study, the mean age was 30 ± 6 years, and the gestational age (GA) at the time of sample collection was 33.2 ± 5.5 weeks. One hundred and fifty (55%) women had no reported comorbidities. The remaining 123 (45%) women were found to have pregnancy-associated comorbidities with hypertensive disorder of pregnancy (25/123 [20.3%]), DIP (69/123 [56.1%]) and diabetes–hypertensive disorder of pregnancy (23/123 [18.7%]).

### 3.2. Association of Serum and Urine Markers of Renal Function Between Women With Normo-, Micro- and Macroalbuminuria

This study compared clinical and renal function parameters between women categorised into normo-, micro- and macroalbuminuric groups (Table 2). Despite there being no significant difference in SCr levels between the three groups, there was a significant difference in sCysC (mg/L) between women with normo- and microalbuminuria and normo- and macroalbuminuria (*p* < 0.05). There was no significant difference observed for the sCysC between the micro- and macroalbuminuria groups. There was no significant difference between the urine creatinine levels of the women with normo-, micro- and macroalbuminuria. There was, however, a significant difference in urinary nephrin and NCR levels between these three groups (*p* < 0.001). The urinary nephrin and NCR increased with increasing urinary ACR levels, and this was significant (*p* < 0.05) (Table 2). There was a significant correlation between urinary, NCR and ACR across the whole (*r* = 0.29, *p* < 0.0001, *N* = 273) and when compared to urine albumin (*r* = 0.24, *p* < 0.0001, *N* = 273) and the combined micro–macroalbuminuria group (ACR ≥ 3 mg/mmol) (*r* = 0.39, *p*=0.006, *N* = 48). Overall, urinary nephrin and NCR increased significantly and comparably across the three categories of albuminuria. There was a significant difference between women with normoalbuminuria and micro- and macroalbuminuria for urinary nephrin, NCR, urinary albumin and serum cystatin C.

### 3.3. Association of Urinary NCR With the Diagnosis of Diabetes or PE During Pregnancy

**Figure 2:**
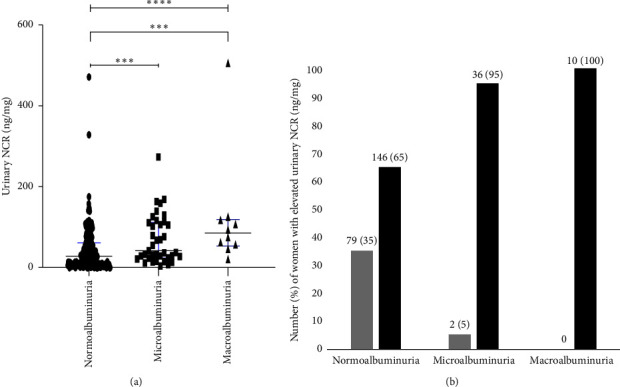
Association of urinary NCR with albuminuria categories: (a) number (percentage) of women with elevated urinary NCR in each of the urinary ACR groups. (b) Normo: < 3 mg/mmol, Micro: 3–30 mg/mmol and Macro: > 30 mg/mmol using urinary NCR cut-off value of < 14 ng/mg for normal nephrinuria (grey bars) and ≥ 14 ng/mg for elevated nephrinuria (black bars). The height of the bar in (b) represents the percentage. ⁣^∗∗∗∗^*p* < 0.0001; ⁣^∗∗∗^*p* < 0.001.

There was a significant association between urinary NCR and urinary ACR categories (Figure 2(a)). A significant difference in urinary NCR was observed between normoalbuminuric and microalbuminuric women and normoalbuminuric and macroalbuminuric women (Figure 2(a)). The percentage of pregnant women with elevated nephrinuria is shown in Figure 2(b). The normal value for urinary nephrin (nephrinuria) in different subgroupings of pregnant women was determined using the ROC-generated cut-off values (Table 3). There was a high proportion of pregnant women showing elevated nephrinuria (NCR > 14 ng/mg). In the full cohort, nephrinuria was detected in 146/225 (65%) women with normoalbuminuria, 36/38 (95%) women with microalbuminuria and 10/10 (100%) women with macroalbuminuria at a cut-off value of 14 ng/mg (Figure 2(b)).

**Figure 3:**
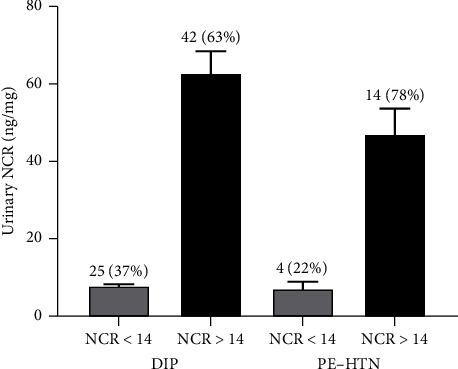
The mean (SEM) levels of urinary NCR and number (percentage) at the top of the bar for women within the normoalbuminuric group who were diagnosed with either a hypertensive disorder (PE/HTN) or diabetes in pregnancy (DIP). These are categorised using urinary NCR (cut-off value of < 14 ng/mg for normal nephrinuria, grey bars, and ≥ 14 ng/mg for elevated nephrinuria, black bars).

Within the group of women with normoalbuminuria demonstrating elevated nephrinuria, 85/225 (38%) showed heterogeneity in their clinical characteristics and comorbidities, 5/225 (2%) had a history of renal disorders, and 135/225 (60%) had no reported comorbidities (Figure 2). This group of normoalbuminuric women is further explored in Figure 3. Of the women with normoalbuminuria who also had elevated nephrinuria [NCR ≥ 14 (ng/mg)], 14/18 (78%) were diagnosed with a hypertensive disorder (PE/HTN) with mean ± SEM (47.1 ± 6.9) of urinary NCR, 42/67 (63%) were diagnosed with DIP with mean ± SEM (62.8 ± 5.9) of urinary NCR, and 2/5 (40%) of nephrinuria women had a history of kidney disease.

### 3.4. Predictive Characteristics of Urinary NCR for Determining Glomerular Injury

The predictive characteristics of urinary NCR for significant albuminuria as an indicator of glomerular injury were explored. The women were grouped according to ACR as the main determinant (normoalbuminuria (ACR< 3 mg/mmol) and micro–macroalbuminuria (≥ 3 mg/mmol)). Table 3(a) compares the urinary nephrin in the full cohort (*N* = 273). The 48 women had albuminuria (ACR ≥ 3 mg/mmol) and 225 women had normoalbuminuria (ACR< mg/mmol). Comparing urinary nephrin between ACR ≥ 3 mg/mmol, *N* = 48, vs. ACR < 3 mg/mmol, *N* = 225, in the full cohort (*N* = 273). Then, further subgrouping women based on the reported comorbidities: micro–macroalbuminuria with comorbidities (ACOM) (ACR ≥ 3 mg/mmol, *N* = 33) vs. women who had normoalbuminuria with no comorbidities (NORM) as a control group (ACR < 3 mg/mmol, *N* = 135) (Table 3(b)**)**, and then women with comorbidities who had ACR ≥ 3 mg/mmol (ACOM), *N* = 33, versus women who had comorbidities but ACR < 3 mg/mmol (NCOM), *N* = 90 (Table 3(c)). A ROC analysis showed that the diagnostic accuracy of urinary NCR for predicting glomerular injury was satisfactory (AUC > 0.7) in the entire cohort. The result showed a *r* sensitivity of 83% but specificity of 48% (Table 3(a)). An improved predictive probability is observed when stratifying for different cut-off values (Tables 3(b) and 3(c)). Overall, urinary nephrin and NCR show moderate predictive probability (AUC: 0.69–0.75). Urinary nephrin and NCR exhibited a low positive predictive value (PPV), but a higher negative predictive value (NPV).

### 3.5. Predictive Characteristics of Urinary Nephrin, NCR and ACR for Determining PE

Using the AUC–ROC analysis, the cut-off value for urinary NCR was 14 ng/mg and significant differences were observed between women who developed PE and who did not develop PE (*p*=0.038). The cut-off value for urinary ACR calculated using ROC analysis for this cohort was ≥ 3.7 mg/mmol, and this compared well with the KDIGO recommended cut-off value for urinary ACR was ≥ 3 mg/mmol. The sensitivity of urinary NCR was found to be higher (92%) than the specificity of 32%, and this was comparable with urinary nephrin (92% and 42%, respectively). The urinary nephrin and NCR exhibited poor PPV (< 15%) but higher NPV (> 97%). Urinary ACR showed 50% sensitivity and 89% specificity to predict glomerular injury of PE. A comparison of the performance of these markers in the entire cohort (*n* = 247) is given in Table 4.

### 3.6. Association of Urinary Nephrin, NCR and ACR With Diagnosis of PE and Diabetes During Pregnancy

**Figure 4:**
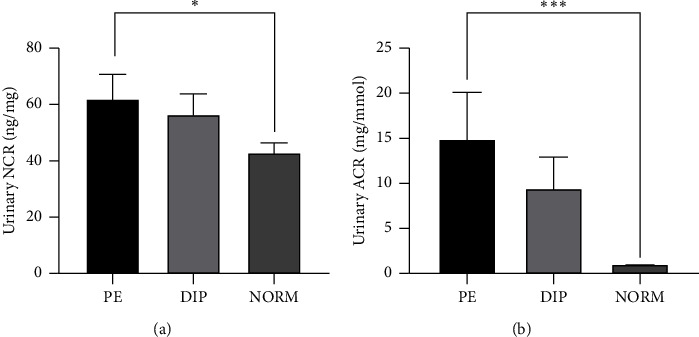
Urinary NCR ng/mg values in pregnant women who developed a pregnancy complication, preeclampsia (PE), diabetes in pregnancy (DIP) compared with women with ACR < 3 mg/mmol and no reported comorbidities (NORM). Values are described as mean (SEM). ⁣^∗^*p* < 0.05, ⁣^∗∗∗^*p* < 0.0001.

Markers of renal function were compared between pregnant women who developed PE and DIP and women with a normal ACR < 3 mg/mmol and no reported comorbidities (NORM) in the full cohort. There was no significant difference between the three groups for the urine creatinine and albumin, or SCr and sCysC between the three groups (*p* > 0.05). There was a significant difference between the mean urinary NCR (61.9 [9.2] versus 56.4 [7.7]) and 42.7 [3.9]) ng/mg, urinary nephrin (81.9 [18.2] versus 57.8 [9.2]) and 36.7 [4.3]) ng/mL and ACR (14.9 [5.4] versus 9.4 [3.7]] and 1.04 [0.05]) mg/mmol. A statistically significant difference was observed between women who developed PE and the normal albumin group (NORM) (*p* < 0.05) (Figures 4(a) and 4(a)). There was no significant difference in urinary NCR between the women who developed DIP and the NORM group.

### 3.7. Comparison of Urinary Nephrin and NCR for the Prediction of PE and DIP With ACR Levels

**Figure 5:**
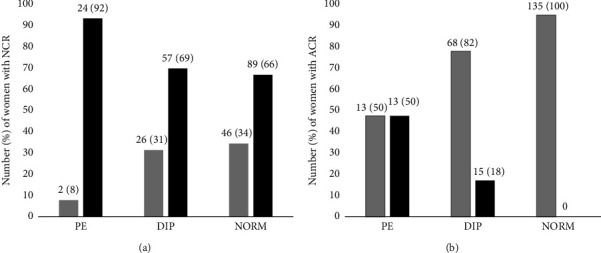
The number (percentage) of women from the full cohort identified using either NCR (a) or ACR (b) who developed PE, women who had DIP and women who did not have reported comorbidity with normoalbuminuria in the full cohort. (a) Normal urinary NCR (grey bars) and high urinary NCR (black bars) at 14 ng/mg cut-off value. (b) Normal ACR (grey bars) and high ACR (black bars) at 3.7 mg/mmol cut-off value in the full cohort. The height of the bar represents the percentage.

The number (percentage) of women who developed PE and DIP was compared to a group of women with normal albuminuria and no comorbidities using NCR < 14 ng/mg as a cut-off. Elevated nephrinuria was detected in 92% of women who developed PE (Figure 5(a)), while using the cut-off values of urinary ACR obtained from ROC (similar to the KDIGO guideline provided cut-off value) (Figure 5(b)), only 13/26 (50%) of women with PE were identified with high albuminuria.

## 4. Discussion

This study investigated the use of urinary nephrin and NCR for determining early glomerular injury in a cohort of pregnant women. The existing guidelines recommend urinary ACR as a standard reference test for the diagnosis and monitoring of glomerular damage [22]. While an elevated ACR provides a strong indicator of advanced glomerular damage, ACR is a less sensitive marker of early glomerular injury. Albuminuria may also be detected in the urine of patients secondary to other pathological conditions [24]. In the context of early glomerular injury, it has been hypothesised that structural damage precedes albumin leakage through the filtration barrier [13]. Therefore, it is thought that the excretion of the structural protein nephrin might precede microalbuminuria, providing any earlier indicator of glomerular damage [13].

Nephrinuria correlated well with albuminuria and other markers of renal function in this cohort of women. Our study found that urinary nephrin, NCR and sCysC significantly increased with an increase in urinary ACR. Urinary nephrin, NCR and sCysC were significantly elevated in women who had micro- and macroalbuminuria (≥ 3 mg/mmol), when compared to women with normoalbuminuria (< 3 mg/mmol) with and without comorbidities (Table 2). While the percentage of pregnant women in the microalbuminuria and macroalbuminuria groups with elevated nephrinuria was high, 95% and 100%, respectively, there was also a high proportion of normoalbuminuric pregnant women that demonstrated elevated nephrinuria (65%) (Figure 2). The presence of diabetes and/or hypertensive disorders during pregnancy may increase the risk of glomerular damage and AKI. Glomerular injury has been associated with podocyte protein loss [25], and previous studies have suggested that podocyte damage is associated with increased sCysC, urinary nephrin and urinary albumin in patients with diabetes [13, 26–29]. Hence, an increased urinary NCR and sCysC may provide a risk predictor of glomerular injury, indicating subclinical damage to the glomerular podocytes preceding leakage of albumin in the urine. To further investigate this finding, the normoalbuminuric group was evaluated for comorbidities that may be involved in kidney injury (Figure 3). Interestingly, within the normoalbuminuric group, 14/18 (78%) women who had a hypertensive disorder and 42/67 (63%) women who had DIP had elevated nephrinuria. Thus, urinary NCR may be a more sensitive indicator than urinary ACR for predicting early glomerular injury.

A recent study conducted by Kostovska et al. on 90 patients with type 2 diabetes determined that 82% of normoalbuminuric patients, 88% of patients with microalbuminuria and 100% of patients with macroalbuminuria had elevated nephrinuria [13]. Numerous studies have demonstrated that nephrinuria may be a sensitive marker for determining glomerular injury in diabetic patients. Jim et al. found that 54% of DM patients with normoalbuminuria and 100% of patients with micro- and macroalbuminuria demonstrated high urinary nephrin [26]. Shahid et al. using ELISA (Eth-Bio, USA) to quantitate urinary nephrin demonstrated an increased urinary nephrin in 81.4% with normoalbuminuria and 100% of DM patients with macroalbuminuria [28]. Interestingly, Kishore, Silambanan and Moorthy also found that nephrin excretion was significantly higher in DM patients with normoalbuminuria [27], suggesting that urinary nephrin [30] may prove to be a more sensitive indicator of early renal dysfunction.

### 4.1. Urinary NCR as a Predictive Marker of Glomerular Injury in PE and DIP

Proteinuria is a common symptom of glomerular injury during pregnancy, and as a consequence, women are routinely monitored for the development of proteinuria. However, its presence is not required for a clinical diagnosis of PE [23]. In the current study, an elevated urinary NCR (nephrinuria) was detected in 92.3% of women who developed PE, 69% of women identified with DIP and 66% of women with an ACR (< 3.0 mg/mmol) and no reported comorbidities. This is in contrast with those reported to have micro- and macroalbuminuria (> 3.7 mg/mmol), detected in 13/26 (50%) women who developed PE and 15/247 (18%) women identified with DIP. Urinary nephrin, NCR and ACR were significantly higher in women who developed PE when compared with women who did not develop PE. Urinary NCR were significantly correlated with urinary ACR in PE (*r* = 0.50, *p*=0.02). Several studies have found urinary nephrin levels to be significantly higher in women who develop PE when compared to their counterparts [31–34].

A recent study among pregnant women revealed the association of elevated urinary nephrin with PE. The finding showed a ninefold increase in urinary nephrin in PE compared to normotensive women [35]. Likewise, a recent review by Kandasamy, Watson and Rudd described the critical role of nephrinuria in the pathogenesis of proteinuria during PE [36], and the authors suggested nephrinuria as an indicator of glomerular injury. In the past, studies have suggested that podocyturia occurs prior to albuminuria, showing that podocyturia can be detected earlier in PE [25, 37]; therefore nephrinuria, may be a more sensitive marker than angiogenic markers particularly for asymptomatic women [38]. Recently, an observational cohort study investigated the association between PE and long-term kidney outcomes [39] and found that women with PE during pregnancy were at increased risk of later developing chronic HTN, declined glomerular filtration rate (GFR: < 60 mL/min/1.73 m^2^) and increased risk of albuminuria when compared to women who did not develop PE. Early detection of renal injury in PE could be a valuable opportunity to diagnose AKI during pregnancy and therefore identify women at risk of developing CKD and may aid in the development of early intervention strategies and reduce kidney disease in later life.

### 4.2. Predictive Performance of Urinary NCR for the Detection of Glomerular Injury in PE and DIP

The predictive characteristics of urinary NCR in this study using a cut-off value of 14 ng/mg showed a sensitivity of 92.3% and specificity of 32.4% for predicting glomerular injury associated with PE. A review showed that urinary nephrin has a high sensitivity and specificity for predicting renal injury in PE in different cohorts [30]. Kostovska et al. found that urinary nephrin had a sensitivity and specificity of 96.7% comparing patients with T2DM and DN [31]. Jim et al. demonstrated a sensitivity and specificity of 57% and 58%, and Yang et al. demonstrated a sensitivity and specificity of 67% and 83%, respectively, for the prediction of PE [32, 40].

Previous studies investigated the sensitivity and specificity of urinary nephrin for predicting glomerular injury in DN patients [30], and another study found that the sensitivity (92.5%) and specificity (76.7%) for the detection of glomerular nephropathy [27]. Kostovska et al. demonstrated, in 90 patients with T2DM (30 known DN and 60 without diagnosed DN) and 30 healthy controls, that urinary nephrin had a higher predictive probability of 96% for patients with DN [13]. Similarly, Jim et al. demonstrated a comparable finding to our study using an ELISA to quantitate urinary nephrin, with a sensitivity of 99% and 46% specificity for the prediction of glomerular nephropathy [26], and a systematic review also found high sensitivity and specificity of urinary nephrin for predicting glomerular injury [30]. Differences in the reported sensitivity and specificity of results between our findings and those reported by others possibly relate to variation in the recruitment of participants with previous studies using strict inclusion and exclusion criteria to allocate high-risk groups and frequency-matched controls and our prospective study including all women presenting for antenatal screening. Other studies included various stages of patients with DN with a longer duration of illness and have comparable sample sizes between groups to determine the predictive probability of urinary nephrin. However, our study recruited pregnant women with no exclusion criteria. Together, these studies support the idea that urinary nephrin may be a more sensitive biomarker for the recognition of early glomerular injury [30] than albuminuria.

### 4.3. Predictive Performance of Urinary NCR for the Detection of Early Glomerular Injury

This study investigated the use of urinary nephrin and NCR as a marker for determining early glomerular injury in a cohort of pregnant women. Existing guidelines recommend urinary ACR as a standard reference test for the diagnosis and monitoring of glomerular damage [22]. While an elevated ACR provides a strong indicator of advanced glomerular damage, ACR is a less sensitive marker of early glomerular injury. Albuminuria may also be detected in the urine of patients secondary to other pathological conditions [24]. In the context of early glomerular injury, it has been hypothesised that structural damage precedes albumin leakage through the filtration barrier [13]. Therefore, it is thought that the excretion of the structural protein nephrin might precede microalbuminuria, providing any earlier indicator of glomerular damage [13, 30].

Urinary NCR appears to have a high sensitivity for the prediction of early glomerular injury of PE relative to urinary ACR. The majority (82%) of the participants in our study were normoalbuminuric; of these, 52% had an elevated nephrinuria; this group had heterogeneous clinical characteristics and comorbidities, all of which may contribute to an increased risk of podocyte damage. Urinary ACR > 3.7 mg/mmol demonstrated a 50% sensitivity and 88.7% specificity in the prediction of PE; in this cohort, only 50% of women who developed PE had albuminuria. Similar observations have been reported by Jim et al. in 91 pregnant women, of whom 78 were in a high-risk group, and the sensitivity and specificity of albuminuria were found to be 36% and 96%, respectively, to predict PE. However, the authors revealed that none of the low-risk women exhibited albuminuria. A recent study by Devanath et al. demonstrated urinary nephrin as a biomarker of early glomerular injury in newly diagnosed hypertensive patients [41]. The urinary nephrin was quantified using a human nephrin ELISA kit (Elabscience Biotech Co. Ltd., Wuhan, Hubei Province, China). The authors reported significantly high urinary NCR in hypertensive patients with normoalbuminuria, and the investigators concluded that urinary nephrin can be used as a marker for early glomerular injury preceding albuminuria [41].

This might lead to speculation that early glomerular injury can occur without proteinuria in the early stages of PE and that early clinical diagnosis and management of PE could prevent glomerular damage associated with hypertensive disorders. Moreover, mild glomerular endotheliosis has been reported in women with pregnancy-associated HTN without proteinuria [42]. Glomerular damage associated with PE often reverses following delivery, coinciding with HTN management and nonprogressive glomerular damage.

## 5. Strengths and Limitations of the Study

The strength of this study is that it is a cross-sectional study that includes a clinically heterogeneous group of pregnant women who may also present with nephrinuria, such as women with HTN, women with PE and GDM. The study also compares urinary NCR with ACR as a comparison marker to predict glomerular injury since the current guidelines used ACR as a marker for glomerular damage and monitoring and management of CKD. Compared to all other studies on nephrinuria to date, the study had a larger sample size of pregnant women, which is another strength. The limitation of this study is that the cross-sectional nature of the study provides the basis for association, rather than causality. We do not know if nephrinuria is the causal mechanism or if early detection of nephrinuria will reliably predict consequent glomerular injury in women with normoalbuminuria. Finally, the reference test urinary ACR may not reveal subclinical glomerular damage and might underscore the specificity of urinary NCR in this study. The other important aspect is the different methods of the ELISA test kit. This study used the human nephrin sandwich ELISA kit with a narrow assay dynamic range (0.157–10 ng/mL), and the previous literature used a competitive ELISA kit with a higher assay dynamic range (31.3–2000 ng/mL); this may account for variation in the cut-off value between the study groups.

## 6. Conclusions and Recommendations

In conclusion, our findings showed that urinary nephrin and NCR could be used to detect early glomerular injury in pregnant women. Urinary NCR was detected in a high percentage of women with normoalbuminuria, suggesting that this may have a role as a marker of early glomerular injury. There was a significant increase in urinary nephrin, NCR and sCysC in pregnant women with micro- and macroalbuminuria compared with those women with normoalbuminuria. There was a significant positive correlation between urinary NCR and ACR. There was a statistically significant difference in urinary nephrin, NCR and ACR between women who developed PE and women who did not develop PE. Our results suggest that urinary NCR holds discriminatory power in identifying women at risk of significant albuminuria as well as those that develop PE among pregnant women.

Further longitudinal prospective studies are required to investigate the utility of NCR for identifying glomerular injury prior to albumin appearing in the urine and investigate the translation of urinary NCR into clinical practice. There is currently no recommended cut-off value for abnormal urinary NCR excretion in different populations and for the prediction of glomerular injury in low- and high-risk pregnant women. Cut-off values for urinary NCR vary across the literature, making it difficult to compare studies. Therefore, establishing clinically useful RIs for urinary NCR among healthy pregnant women would be of considerable clinical value.

## Figures and Tables

**Table 1:** Maternal clinical characteristics and pregnancy outcomes.

Variables	Count/mean ± SD	Percent (%)
Maternal age at sample collection time (years)	30.0 ± 6.0	
GA at sample collection time (weeks)	33.2 ± 5.5	
First trimester	0	0
Second trimester	38	14
Third trimester	235	86
Patients with no reported comorbidities	150	55
Patients with reported comorbidities	123	45
Hypertensive disorder of pregnancy		
Preeclampsia (PE)	16	13
Hypertension (HTN)	9	7.3
Diabetes in pregnancy		
Gestational diabetes (GDM)	56	45.5
Type 2 diabetes mellitus (T2DM)	9	7.3
Type 1 diabetes mellitus (T1DM)	4	3.3
Diabetes–hypertensive disorder of pregnancy		
T2DM–HTN	4	3.3
GDM–HTN	9	7.3
GDM–PE	7	5.7
T2DM–PE	3	2.4
Chorioamnionitis	1	0.8
History of renal problem	5	4.1

*Note:* Data are given as count or mean ± SD or %.

Abbreviations: GA: gestational age; SD: standard deviation.

**Table 2:** Association of maternal urinary and serum indices with albuminuria categories.

Variables	Normo (*N* = 225)	Micro (*N* = 38)	Macro (*N* = 10)	*p* value
*Urine markers (n = 273)*				
Urinary nephrin (ng/mL)	9.7 (5.4–68)	34.4 (14–91)^1^	82.1 (30–152)^2^	< 0.001
Urinary NCR (ng/mg)	27.7 (10–64)	44.7 (26–115)^1^	86.3 (56–122)^2^	< 0.001
Urine albumin (mg/L)	5.8 (3.2–11.4)	63.8 (21.2–149)^1^	525 (246–921)^2^	< 0.001
Urine creatinine (mmol/L)	6.4 (3.6–11.5)	7.4 (4.8–13.4)	6.5 (4.1–11.2)	0.508

*Serum markers (n = 271)*				
Serum cystatin C (mg/L)	0.96 (0.8–1.2)	1.12 (0.9–1.8)^1^	1.34 (0.9–2.1)^2^	0.007
Serum creatinine (μmol/L)	44 (39–51)	43 (40–53)	52 (42–83)	0.085

*Note:* Values are reported as median (IQR). Normoalbuminuria (< 3.0 mg/mmol), microalbuminuria (3.0–30 mg/mmol) and macroalbuminuria (> 30 mg/mmol) for urinary nephrin (ng/mL) (*χ*^2^ = 17.97), urinary NCR (ng/mg) (*χ*^2^ = 21.88) and sCysC (mg/L) (*χ*^2^ = 9.9) (Kruskal–Wallis H test with Bonferroni post hoc analysis; *p* < 0.05).

^1^Significant difference between normoalbuminuria versus microalbuminuria (*p* < 0.05).

^2^Significant difference between normoalbuminuria versus macroalbuminuria (*p* < 0.05).

**Table 3:** Sensitivity, specificity and reliability of urinary nephrin for determining glomerular injury in (A) all pregnant women, (B) normal control group and (C) those with comorbidities.

Biomarker	Sensitivity (%)	Specificity (%)	Negative PV (%)	Positive PV (%)	Cut-off	AUC (95% CI)	*p* value
*A. Comparing urinary nephrin between ACR ≥ 3 mg/mmol, N = 48, vs. ACR < 3 mg/mmol, N = 225, in the full cohort*							
Urinary NCR	83	48	93	26	24	0.71 (0.63–0.78)	< 0.001
Urinary nephrin	79	57	93	28	17	0.69 (0.61–0.77)	< 0.001

*B. Comparing urinary nephrin (ACOM ACR ≥ 3 mg/mmol, N = 33, vs. NORM ACR < 3 mg/mmol, N = 135)*							
Urinary NCR	97	36	98	27	14	0.74 (0.65–0.82)	< 0.001
Urinary nephrin	82	60	93	35	16	0.74 (0.65–0.83)	< 0.001

*C. Comparing urinary nephrin between women with comorbidities (ACOM: ACR ≥ 3 mg/mmol, N = 33, vs. NCOM: ACR < 3 mg/mmol, N = 90)*							
Urinary NCR	88	48	92	37	22	0.75 (0.65–0.84)	< 0.001
Urinary nephrin	85	54	91	40	17	0.72 (0.63–0.82)	< 0.001

Abbreviations: AUC, area under the curve; CI, confidence interval; PV, predictive value.

**Table 4:** Predictive performance of urinary nephrin and ACR for PE (PE: *N* = 26 vs. non-PE: *N* = 247).

Diagnostic performance	Urinary NCR	Urinary nephrin	Urinary ACR
Sensitivity (%)	92	92	50
Specificity (%)	32	42	89
Negative predictive value (%)	98	98	94
Positive predictive value (%)	13	14	32
Optimal cut-off value	14	9	4
AUC (95% CI)	0.63 (0.52–0.73)	0.68 (0.58–0.78)	0.71 (0.59–0.82)
*p* value	0.038	0.003	0.001

Abbreviations: AUC, area under the curve; CI, confidence interval.

## Data Availability

The data that support the findings of this study are available from the corresponding author upon reasonable request.
